# Socioeconomic disparities in healthcare utilization under universal health coverage: evidence from Dubai household health survey

**DOI:** 10.1186/s12939-022-01691-8

**Published:** 2022-06-25

**Authors:** Shreena Malaviya, David Bishai, Meenu Mahak Soni, El Daw Suliman

**Affiliations:** 1 Johns Hopkins Bloomberg School of Public Health, Johns Hopkins University, Baltimore, MD USA; 2 Department of Population, Family and Reproductive Health, Johns Hopkins Bloomberg School of Public Health, Johns Hopkins University, 615 N Wolfe St, Baltimore, MD 21205 USA; 3 Dubai Health Insurance Corporation, Dubai Health Authority, Dubai, United Arab Emirates

**Keywords:** System of health accounts, UHC, Healthcare utilization, Socioeconomic equity

## Abstract

**Background:**

In 2013, Dubai implemented the Insurance System of Advancing Health in Dubai (ISAHD) law which required mandatory health insurance for all residents of Dubai effective in 2016. This study compares the effect of the ISAHD on the utilization and out-of-pocket (OOP) expenditures for low and high socio-economic status sub-groups.

**Methods:**

The study used the 2014 and 2018 Dubai Household Health Survey (DHHS) a representative survey of Dubai stratified as: 1) Nationals; 2) Non-nationals in households; 3) Non-nationals in collective housing; and 4) Non-nationals in labor camps. The probability that each household would have expenditures was calculated, then multiplied by a weighted estimate of the average total OOP expenditure.

**Results:**

Overall Dubai’s health spending rose from 12.8 billion AED (3.4 billion US $) in 2014 to 16.8 billion AED (4.6 billion US $) in 2017. Concurrently, the OOP share in total health spending in Dubai fell from 25% in 2014 to 13% in 2017. From 2014 to 2018, there were increases in the utilization of inpatient, outpatient and discretionary services for all groups except non-nationals living in camps. In 2018, nationals spent a total of 1064.65 AED, non-nationals in households spent 675.01 AED, collective households spent 82.35 AED, and labor camps spent 100.32 AED out-of-pocket per capita for healthcare expenditures. During and after the implementation of ISAHD, there was a substantial growth in the OOP expenditure per capita for nationals and non-nationals in households due to increased utilization. OOP spending did not rise for the lower-income non-National households.

**Conclusion:**

Dubai has been successful in reducing the household share of OOP expenditures by shifting the financial burden to government and employers. Emiratis and expatriate households increased their health service utilization after ISAHD but blue-collar workers did not. Remaining non-financial barriers to care for Dubai’s blue-collar workers must be identified and addressed.

## Key messages

- Mandatory Universal Health Insurance in Dubai was started in 2013 and has reduced out of pocket health spending but by 2018 it did not increase health utilization in lower socio-economic status migrant groups
- Between 2014 and 2018 overall spending per capita and the utilization of outpatient and inpatient health services increased
- Inpatient health utilization doubled and out of pocket spending increased five fold in n Emirati nationals
- Inpatient health utilization did not increase and out of pocket spending increased by 8% in Non-Nationals living in Collective Housing

## Background

Universal Health Coverage (UHC) aims to reduce financial burdens and impoverishment due to health expenditures while increasing access to important health services for all people [1]. The path to UHC requires universal enrollment in a system for financial protection as well universal access to appropriate promotive, preventive, curative, and rehabilitative health services of high-quality. Universal health coverage can prevent health-related financial shocks that can push households into poverty [1, 2].

The emirate of Dubai, one of the seven emirates of the United Arab Emirates, has advanced significantly on their path to achieving UHC. The health sector of the Emirate of Dubai accounted for 4.6% of the GDP in 2018. The sector along with its free zones (special economic zones or regions in Dubai set up with the objective of offering tax concessions and customs duty benefits to expatriate investors), excluding Dubai Health Care City Authority (DHCA), is regulated and overseen by the Dubai Health Authority (DHA), a public organization involved in strategic oversight of the sector and enhancing private sector engagement. DHA carries out regulation, licensing, and management of public and private facilities as well as the financing of the public facilities. The Ministry of Health and Prevention (MOHAP) and Dubai Health Care City Authority (DHCA) also play a role in oversight over a limited number of facilities compared to the DHA.

The population of Dubai was an estimated 4.67 million in 2018, of whom 1.47 million were holders of a Dubai visa but who lived in other emirates. Geographically, the inhabitant population of Dubai consists of 8% UAE nationals and 92% expatriates. There are nearly 16 million tourists that visit Dubai each year based on data from 2018. Approximately 60% of the population consists of low-income blue-collar workers. Blue collar expatriate workers have shorter stays than white collar expatriates and they immigrate during high demand in the construction and infrastructure sectors, and emigrate when the projects are completed.

Before the healthcare financing reforms in 2013, the citizens of UAE (Emiratis) residing in Dubai could receive free healthcare from government providers. They also received publicly provided health insurance that largely covered privately provided services. Prior to the reforms nearly all non-Emiratis residents in Dubai would be charged for healthcare services except for emergency, maternal and child care [3]. The expatriates could either pay out-of-pocket for healthcare or be covered through individually or employer purchased health insurance plans. They also had the option to purchase a health coverage plan from the federal level of UAE, that entitled beneficiaries to access medical services at all Ministry of Health facilities for minimal fees [3]. Thus, there were marked differences in the coverage and access to healthcare between different population sub-groups primarily determined by their citizenship status and income levels [4, 5].

To counter the growing inequality in access and affordability of healthcare, Dubai reformed its health system with policies designed to achieve Universal Health Coverage (UHC). In November 2013, the government of Dubai passed the Mandatory Health Insurance law – Law 11, known as the Insurance System of Advancing Health in Dubai (ISAHD), requiring mandatory health insurance enrollment for all residents of Dubai effective in 2016. The law was implemented in stages across Dubai to reduce disruptions to the healthcare system. The period before 2013 can be considered the pre-ISAHD phase, 2013–2015 would be the implementation phase of ISAHD, and 2016 onwards is the post- implementation phase of ISAHD.

ISAHD placed a mandate requiring health insurance on all employers and employees. It was a requirement for all work visas issued by Dubai government. This model ensured that Dubai’s residents had financial access to healthcare and protects the financing system from adverse risk selection, where people who think they are at low-risk could opt out of buying insurance. The private insurance contracts offer patients the choice to seek treatment with government or private health care providers. In addition, the government of Dubai provides health insurance for all its expatriate employees and their families (Husband/wife and up to 3 children under the age of 21 years) through the ENAYA Program, and for all the citizens of Dubai through the SAADA program. One measure of the success of the ISAHD law could be defined by the decrease in the out-of-pocket expenditure (OOP) by the population. Another measure of success would be an increase in the utilization of health services. A third measure of success would be comparative parity in health care utilization trends across social groups, noting that there might be age-based differences in needs when comparing Emirati Nationals to expatriate blue collar and white-collar workers.

## Methods

### Population selected for analysis

The population of health system users of Dubai can be classified as follows:

1. UAE Nationals (Emiratis) in the Emirate of Dubai
2. Non-nationals (Expatriates) with employment visas from Dubai and residence inside Dubai
3. Non-nationals (Expatriates) with employment visas from Dubai and residence outside Dubai
4. Tourists who visit Dubai

The Dubai Statistics Centre considers only the first two groups as part of Dubai’s population. However, according to the law, government agencies and private employers in Dubai are mandated to offer healthcare coverage to all employees with Dubai employment visas regardless of their geographical residence. Thus, for the purposes of this study, the first three groups will be considered. We can further segregate the non-nationals based on their type of residence – households, collective housing and labor camps. Non-nationals residing in labor camps represent a less privileged social class than those living in households and those living in collective housing have intermediate social status. Health spending by tourists is not included in this study as the population of short-term tourists varies seasonally.

### Data - Dubai household health survey (DHHS) 2014 and 2018

The Dubai Household Health Survey (DHHS) is the largest comprehensive household survey of healthcare and health issues carried out in the Emirate of Dubai. The survey provides a statistically accurate and representative outlook of key health and healthcare variables across the entire population of Dubai. It was first conducted in 2009, and repeated in 2014 and 2018.

The surveys of 2014 and 2018 were based on a multi-stage stratified cluster sample. The sampling was designed so that after weighting it would be representative of the four sub-populations: UAE citizens, Non-nationals living in households, Non-nationals living in collective housing, and Non-nationals living in labor camps. Surveyors personally visited these randomly selected households to obtain detailed information on issues ranging from household health expenditure, and access to health services to questions on exercise levels, dietary habits, lifestyle diseases, mental health, and a detailed module on the use of public and private health services in Dubai. The 2018 survey was designed by DHA and the logistics and field work was led by the Dubai Statistics Center (DSC), and had a response rate of 96%. The design and methodology of the survey were adopted from those used in the World Bank’s Living Standards Measurement Surveys (LSMS), the World Health Organization’s World Health Surveys (WHS) and the US Center for Disease Control’s National Health Interview and Examination Surveys (NHIES).

Importance weights were assigned by Dubai Statistics Center because UAE citizens were oversampled. After weighting, the sample was representative of a geographical population of 3.2 million Dubai residents as of 2018. The sample size for 2018 was a total of 9630 persons in 2200 housing units of whom 5665 were UAE citizens, 2342 were Non-Citizens in Households, 1335 were Non-Citizens in collective housing, and 288 were Non-Citizens in labor camps. The samples in 2014 and 2018 included a total of 3271 and 2200 separate household units respectively. The survey was sanctioned by the institutional review board of the Dubai Health Authority.

The surveyors each received extensive training in the collection of self-reported expenditure data and interviewed a person in the household who is 15 years and above and most knowledgeable about recent medical utilization. After collecting a household roster and basic demographics for each household member, the surveyor asked whether each household member had had any outpatient utilization in the last 30 days, made any discretionary purchases of medical supplies or over the counter medicines (mentioning blood pressure cuffs, blood sugar monitors, orthopedic supplies, medicines, etc.) in the last 30 days and whether each household member had had an overnight inpatient stay in the last 12 months. For households where more than one member had experienced outpatient utilization in the last 30 days, an individual member was selected at random and details of their medical events were collected to investigate the total of out of pocket spending for various categories of discretionary spending, outpatient spending and inpatient spending, after adjusting for the appropriate weights. All household members who had overnight inpatient stay in the last 12 months were asked to fill an inpatient module questionnaire.

### Data - system of health accounts 2012–2017

The results from the published System of Health Accounts (SHA) from 2012 to 2017 were used for triangulation to ensure the OOP expenditure estimates from the survey data were accurately captured for the population defined [6].

### Analysis

In our analysis of DHHS we calculated the probability that each of the 4 categories of household would have any discretionary, or any outpatient, or any inpatient out of pocket spending. For outpatient spending the household survey included the number of outpatient encounters experienced in a 30-day period and the number of hospital admissions experienced in a 12-month period. Then for each type of household the probability of any spending was multiplied by a weighted estimate of the annual average total out of pocket expenditure for households who incurred that type of event. Estimates were adjusted for the incidence of multiple outpatient visits in a month. Only 5 households reported greater than one hospitalization per month, so this adjustment had a negligible effect on estimates of hospitalization incidence and costs. Health care spending data can be dominated by outliers that could dramatically skew estimates of average expected spending in small samples. This was found to be true of the DHHS health spending data. Consequently, all calculations of average health spending excluded outliers above the 99th percentile to reduce the skewness of the data.

Estimates of outpatient and discretionary spending in the last 30 days were annualized to offer estimates of total annual out of pocket spending for each of the four types of households. Finally, total annual spending for each type of household in 2018 was multiplied by estimates of the proportion of these households in the population of Dubai in 2014 based on government data showing that at this time there were 8% UAE citizens, 42% UAE non-nationals living in households, 11% UAE non-nationals living in collective housing, and 39% living in labor camps.

The findings on out of pocket spending for both 2014 and 2018 were used to linearly interpolate to 2015, 2016 and 2017 by adjusting for population growth of the total population and assuming that the proportion of each type of household remained constant. Costs from different years were deflated from and inflated, using the consumer price index so that costs are expressed in real AED with a baseline of 2014.

## Results

**Fig. 1:**
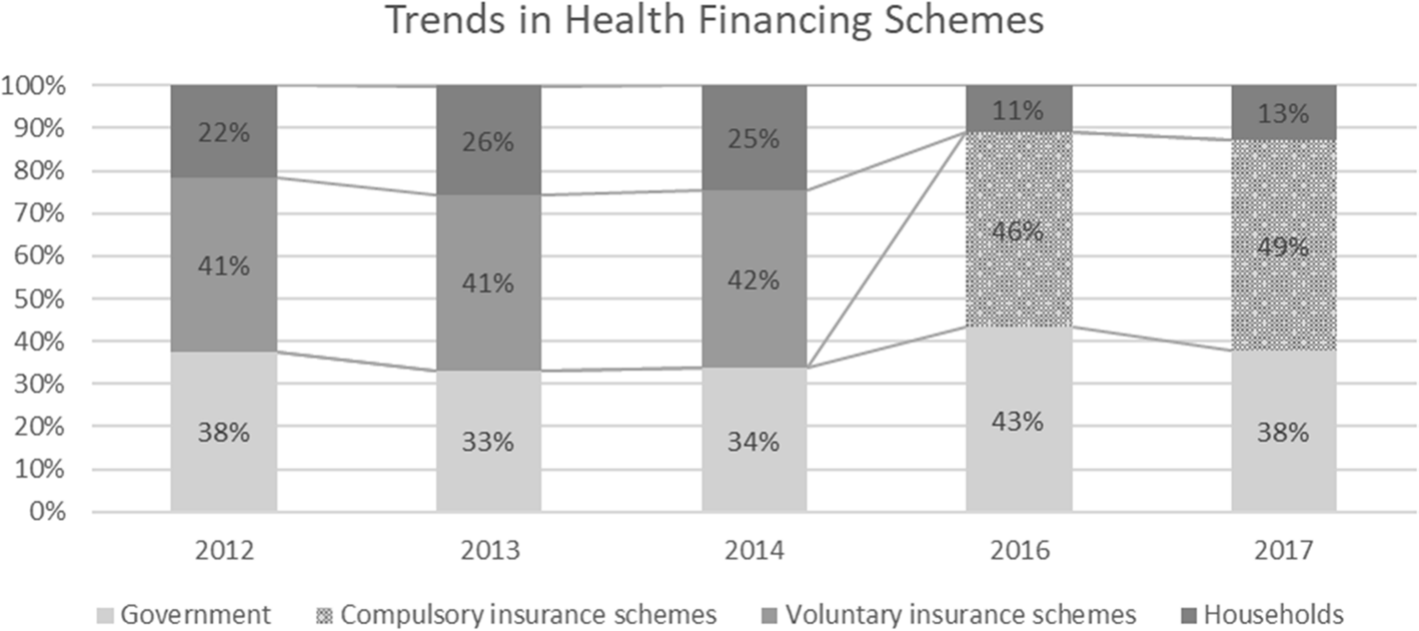
Trends in Health Financing Schemes, Dubai (2012–2017)

We can observe the impact of the ISAHD implementation on the household expenditure and healthcare utilization between the population sub-groups as viewed through the System of Health Accounts in Fig. 1. The figure shows how the share of healthcare financing has evolved between different sources – government-funded healthcare, voluntary insurance schemes, compulsory insurance schemes and OOP expenditure by households. We observe that with the introduction of ISAHD, voluntary insurance schemes are replaced completely by compulsory insurance schemes. Overall, there has been a significant decrease in the total household OOP expenditure by households for the entire population after the implementation of ISAHD (6, Fig. 1).

Table 1 shows the changes in health utilization between 2014 and 2018 for the four demographic groups in the study. Utilization probabilities are typically higher for Emirati nationals. It is important to remember that Emirati nationals include a significantly higher proportion of seniors than non-Emirati households. There were increases in utilization of services for almost all groups for all types of health care utilization. Notable exceptions were the lack of any increase in inpatient utilization for Non-Emiratis living in collective housing.

**Table 1:** Probability of Healthcare Utilization in 2014 and 2018

Group	Year	Any Discretionary Utilization [1]	Any Outpatient Utilization in Month [2]	Any Inpatient Utilization in 12 Months [3]
**Emirati Nationals**	2014	0.8%	10.7%	3.6%
	2018	2.2%	18.3%	6.9%
***Non-Emiratis living in***				
**Households**	2014	0.6%	7.4%	2.3%
	2018	3.3%	14.8%	4.1%
**Collective Housing**	2014	0.2%	2.4%	0.7%
	2018	0.3%	4.3%	0.7%
**Labor Camps**	2014	0.1%	4.6%	1.0%
	2018	0.0%	14.6%	3.1%

[1] Z-statistic =1.83 (Pr = 0.07) for 2014 difference between Emiratis and non-Emiratis in Collective Housing

[2] Z-statistic =8.47 (Pr = 0.00) for 2014 difference between Emiratis and non-Emiratis in Collective Housing

[3] Z-statistic =4.75 (Pr = 0.00) for 2014 difference between Emiratis and non-Emiratis in Collective Housing

Table 2 estimates the total annual spending showing that in 2014, the total OOP expenditure per capita for nationals (AED 182.83) and non-nationals living in households (AED 219.15) was higher than that of the other two groups. The relative differences were driven by statistically significantly higher probabilities of outpatient and inpatient utilization for Emirati nationals than Non-Emiratis in collective housing (See Footnotes to Table 1). Given their higher incomes, the share of the total OOP as part of their income was lower.

**Table 2:** Total OOP per capita (AED) for Different Healthcare Services

Year	Population Sub-Group	Total OOP Discretionary Spending per capita	Total OOP Outpatient Spending per capita	Total OOP Inpatient Spending per capita	Total OOP Spending per capita	Total OOP as Share of Income
2014	Nationals	46	134	3	183	0.03%
	Non-Nationals in Households	42	171	7	219	0.07%
	Non-Nationals in Collective Housing	15	60	0	76	0.08%
	Non-Nationals in Labor Camps	1	39	0	40	0.18%
2018	Nationals	461	484	119	1065	0.18%
	Non-Nationals in Households	281	348	47	675	0.21%
	Non-Nationals in Collective Housing	3	79	0	82	0.09%
	Non-Nationals in Labor Camps	0	82	19	100	0.44%

**Fig. 2:**
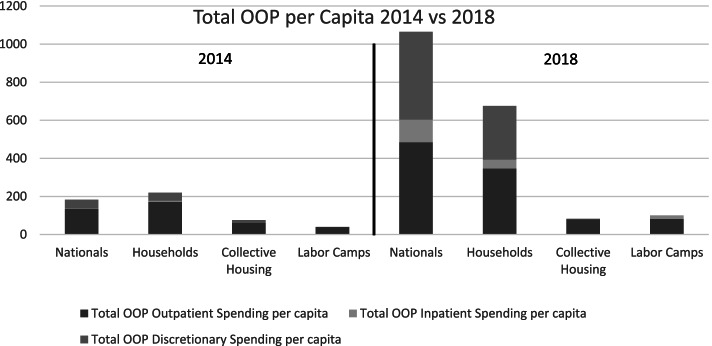
Total OOP per capita (AED) comparison for each population group - 2014 vs 2018

Following full implementation of the health insurance reforms, in 2018, nationals spent a total of 518 million AED, non-nationals in households spent 1496 million AED, collective households spent 63 million AED, and labor camps spent 118 million AED out-of-pocket for healthcare expenditures. We observe a substantial increase in the OOP expenditures with the highest growth in the Emirati nationals (482% growth from 183 to 1065 AED) and the lowest growth in the group of non-nationals living in collective households (8% growth from 76 to 82 AED). Assuming income has remained relatively stable since 2014, the share of the total OOP as part of their income substantially increased for all groups with the exception of collective households (+ 1% growth). The non-nationals in labor camps increased their total OOP spending capita from 2014 to 2018 by 60% and thereby increasing the share of their income spent on OOP healthcare from 0.18% in 2014 to 0.44% in 2018 (Table 2, Fig. 2).

**Fig. 3:**
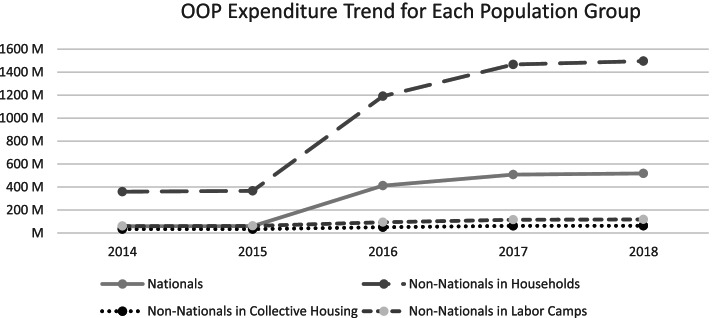
Total OOP Expenditure (Million AED) Trend for Each Population Group

**Fig. 4:**
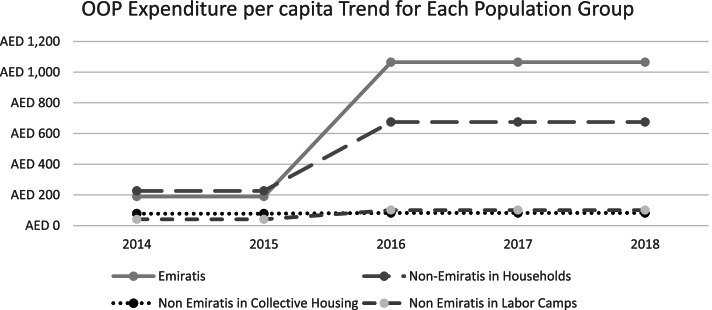
Total OOP Expenditure per capita Trend for Each Population Group

Over the course of implementation of ISAHD, we observed a substantial growth in aggregated total OOP expenditure for nationals and non-nationals in households. However, the growth for the other two demographic groups was attenuated in comparison (Table 3, Fig. 3). We also observed that the growth of OOP expenditure per capita was higher for the Nationals than the Non-Nationals. As seen before, growth for the lower-income households was negligible (Fig. 4).

**Table 3:** Total OOP Expenditure (Million AED) from 2014 to 2018

Population	2014	2015	2016	2017	2018
Nationals	55 M	56 M	412 M	508 M	518 M
Non-Nationals in Households	336 M	343 M	1190 M	1466 M	1496 M
Non-Nationals in Collective Housing	31 M	32 M	51 M	62 M	64 M
Non-Nationals in Labor Camps	56 M	57 M	94 M	116 M	118 M

After the implementation of the ISAHD, the utilization and expenditure patterns for different healthcare services varied between the population sub-groups. Previously, all groups spent more on outpatient care than inpatient and discretionary. By 2018, Nationals and non-nationals were spending more on discretionary and outpatient services than inpatient services. However, non-nationals in collective households had increased the proportion of their expenditure on outpatient services and decreased the proportion of expenditure on discretionary services. The group living in labour camps had increased their proportion of their OOP spending on inpatient services (Table 4).

**Table 4:** Comparison of health service Total OOP spending and its proportional makeup - 2014 vs 2018

Year	Population sub-group	Total OOP Expenditure per capita (AED)	Share of Discretionary Expenditure	Share of Outpatient Expenditure	Share of Inpatient Expenditure
2014	Nationals	183	23%	69%	2%
	Non-Nationals in Households	219	18%	73%	3%
	Non-Nationals in Collective Housing	76	19%	75%	0%
	Non-Nationals in Labor Camps	40	2%	91%	0%
2018	Nationals	1065	40%	43%	10%
	Non-Nationals in Households	675	39%	48%	6%
	Non-Nationals in Collective Housing	82	4%	89%	0%
	Non-Nationals in Labor Camps	100	0%	76%	17%

### Limitations

There are a few limitations associated with this analysis. Firstly, the data collected through the DHHS is self-reported and does not allow for the collection of OOP expenditure estimates for non-traditional medicines. The DHHS oversampled Emirati Nationals and used sample weights to ensure better precision for estimates from this population. Health spending estimates are sensitive to outliers with extreme values of utilization and spending. To limit this distortion our analysis censored observations of expenditure above the 99%ile.

In addition, there are possible cultural and language barriers that prevent accurate collection of data from blue-collar workers. The reports in the DHHS were validated against electronic claims processed by the Dubai Health Authority and published in the official System of Health Accounts.

## Conclusions

The main goal of ISAHD was to ensure that every resident of Dubai would have access to affordable health care and prevent catastrophic health expenditure. In addition, ISAHD was implemented with a goal of leading health improvement for all the residents, especially for low-income residents. By 2016, all of the residents were covered under government or private insurance. Compared to 2014 when household OOP expenditure accounted for 25% of healthcare spending, Dubai has been successful in reducing the out of pocket share to 12% by 2018 which is comparable with most OECD countries. By comparing the 2018 estimates to previous SHA studies [6–8], we can see that the burden of healthcare costs has shifted more from households to government and employers.

As a proportion, most of the out of pocket health care spending was on outpatient care and discretionary care. For Emiratis and expatriate households, the utilization of health services increased after ISAHD and remained relatively stable afterwards. The increase in utilization is primarily attributed to the purchase of medical goods as a result of the higher insurance coverage. The presence of insurance lowered the effective out of pocket prices for medical goods and simultaneously increased utilization as well as total out of pocket spending.

The insurance-induced changes for Emiratis and expatriate white collar households, in medical utilization post-implementation of ISAHD corroborates the experimental results of the RAND experiment and the results of the Oregon Health Insurance experiment conducted in the US that showed how more insurance coverage stimulates more utilization [9, 10].

The response to improved health insurance financial coverage for non-nationals in collective households and labor camps is strikingly different from the more well-off groups in the population. Collective households and people in expatriates in labor camps did not increase their per capita use of health services. We confirmed internal reports validating increased coverage of new blue-collar workers enrolled in coverage based on the number of new insurance contracts. So, there is evidence that the mandated coverage required by the ISAHD was enforced. For some other reason these groups did not show the typical increase in utilization after receiving health insurance.

Dubai has one of the highest rates of population migration largely due to blue-collar workers [11] who have relatively lower incomes and are less socially connected to resources required to address health needs [12, 13]. Migrant populations are often selectively healthier than the populations they emerge from [14]. However, migrants’ diminished social position in their host country can produce challenges in health care seeking, making them more likely to use urgent and emergency services and less likely to use outpatient specialty care [15]. Similar to other countries, many blue-collar migrant workers in the Middle-East have limited access to health resources [12–15]. Studies of Middle-Eastern immigrants have found that migrant workers from poorer groups are at a high risk of mental illness and occupational injuries due to their living and working conditions [16–22]. Occupational injuries are especially high for migrant construction workers [18, 19]. They are also at high risk for family separation, acculturative stress and discrimination that can negatively impact their physical, emotional, and social health [13].

With the implementation of ISAHD and the focus on employer health insurance, it was expected that the reform would remove the financial barriers to healthcare experienced by blue-collar workers and thereby increase their access and utilization of health services. However, our study did not find a dramatic increase in health care utilization. One unlikely possibility, is that due to a healthy migrant effect and their relative youth the expatriate blue collar workers simply did not need much health care services to begin with and so having better insurance did not induce an increase in utilization. Another possibility is that workers’ individual barriers apart from financial cost that prevents them from seeking healthcare. These include obstacles in scheduling, transport, and logistics, cultural and language barriers, health literacy, and coverage awareness [23–29]. Given these factors, it is possible that workers are not aware they have treatable conditions, and/or are hesitant to seek care because they do not understand the resources and health coverage available to them [23–29]. They may also perceive low job security and worry that they will be sent back to their country if their employer suspects they have an illness [30]. The results of this study are consistent with the literature that documents lower utilization of healthcare services by migrant workers, but the data cannot confirm whether the reason is better health or worse non-financial obstacles to utilization or both [23–30].

If the intent of ISAHD was to extend a safety net of effective health care coverage to all residents and thereby to improve their health outcomes, then understanding the remaining barriers to care for Dubai’s blue-collar workers should become a priority [31]. Future healthcare reforms should focus on mitigating the barriers faced by workers in accessing health care. Reforms should focus on understanding patterns of utilization in migrant workers that have recently arrived by comparing them to migrants who have been in the country for a longer period [27]. Interventions could include government and employer organized programs in cultural competency for providers and outreach in multiple languages to raise health literacy, increase awareness of health coverage, and increase logistical access to healthcare providers [32–36].

## Data Availability

The datasets used and analyzed during the current study were received from the Dubai Statistics Center. They are available from the corresponding author on reasonable request.

## Abbreviations

AED: United Arab Emirates Dirham
DHA: Dubai Health Authority
DHCA: Dubai Health Care City Authority
DHHS: Dubai Household Health Survey
DSC: Dubai Statistics Center
ISAHD: Insurance System of Advancing Health in Dubai
MOHAP: Ministry of Health and Prevention
OOP: Out-of-pocket
SHA: System of Health Accounts
UAE: United Arab Emirates
UHC: Universal Health Coverage
